# Exploiting xylan as sugar donor for the synthesis of an antiproliferative xyloside using an enzyme cascade

**DOI:** 10.1186/s12934-019-1223-9

**Published:** 2019-10-10

**Authors:** Manuel Nieto-Domínguez, José Alberto Martínez-Fernández, Beatriz Fernández de Toro, Juan A. Méndez-Líter, Francisco Javier Cañada, Alicia Prieto, Laura I. de Eugenio, María Jesús Martínez

**Affiliations:** 1 Biotechnology for Lignocellulosic Biomass Group, Centro de Investigaciones Biológicas (CIB-CSIC), c/Ramiro de Maeztu 9, 28040 Madrid, Spain; 2 NMR and Molecular Recognition Group, Centro de Investigaciones Biológicas (CIB-CSIC), c/Ramiro de Maeztu 9, 28040 Madrid, Spain

**Keywords:** Endoxylanase, β-Xylosidase, Response surface methodology, Transxylosylation, Antiproliferative

## Abstract

**Background:**

Currently, industrial societies are seeking for green alternatives to conventional chemical synthesis. This demand has merged with the efforts to convert lignocellulosic biomass into value-added products. In this context, xylan, as one of main components of lignocellulose, has emerged as a raw material with high potential for advancing towards a sustainable economy.

**Results:**

In this study, the recombinant endoxylanase rXynM from the ascomycete *Talaromyces amestolkiae* has been heterologously expressed in *Pichia pastoris* and used as one of the catalysts of an enzyme cascade developed to synthesize the antiproliferative 2-(6-hydroxynaphthyl) β-d-xylopyranoside, by transglycosylation of 2,6-dihydroxynaphthalene. The approach combines the use of two fungal xylanolytic enzymes, rXynM and the β-xylosidase rBxTW1 from the same fungus, with the cost-effective substrate xylan. The reaction conditions for the cascade were optimized by a Central Composite Design. Maximal productions of 0.59 and 0.38 g/L were reached using beechwood xylan and birchwood xylan, respectively. For comparison, xylans from other sources were tested in the same reaction, suggesting that a specific optimization is required for each xylan variety. The results obtained using this enzyme cascade and xylan were similar or better to those previously reported for a single catalyst and xylobiose, an expensive sugar donor.

**Conclusions:**

Beechwood and birchwood xylan, two polysaccharides easily available from biomass, were used in a novel enzyme cascade to synthetize an antiproliferative agent. The approach represents a green alternative to the conventional chemical synthesis of 2-(6-hydroxynaphthyl) β-d-xylopyranoside using a cost-effective substrate. The work highlights the role of xylan as a raw material for producing value-added products and the potential of fungal xylanolytic enzymes in the biomass conversion.

## Introduction

Plant biomass is the main renewable resource in biosphere and it is basically composed of two polysaccharides, cellulose and hemicellulose, together with lignin, an aromatic heteropolymer [1]. Xylan is the most abundant hemicellulose and the second source of organic carbon on Earth, only preceded by cellulose. It is a branched heteropolysaccharide formed by a backbone of β-(1,4)-linked xylopyranoses and different side chains, whose frequency and nature depend mostly on the plant source [2]. The natural degradation of xylan comprises mainly two glycosyl hydrolases: endo-β-1,4-xylanases (EC 3.2.1.8) and β-xylosidases (EC 3.2.1.37). The first ones cut internal linkages of the polysaccharide main chain releasing soluble oligosaccharides known as xylooligosaccharides (XOS), which are attacked from the non-reducing end by β-xylosidases to be depolymerized to xylose. However, as a consequence of its complexity, the total degradation of xylan requires the coordinated action of auxiliary enzymes to remove the side chains [3].

Due to its abundance and low cost, the exploitation of xylan is considered a major goal for bioindustry. Currently, this heteropolysaccharide is a raw material in the production of second-generation bioethanol, but it can also be a source of other value-added products such as antioxidants, biomaterials or xylosides [4, 5]. Xylosides are a specific group of glycoconjugates formed by a non-sugar moiety attached to one or several units of xylose. This definition comprises several compounds with pharmacological and food interest [6, 7]. An interesting example is 2-(6-hydroxynaphthyl) β-d-xylopyranoside (DHNX), a glycoconjugate with clinical potential that interferes in the biosynthesis of heparan sulfate, acting as selective inhibitor of the growth of tumor cells both in vitro and in vivo [8, 9].

Xylosides can be obtained mainly by chemical and enzymatic synthesis, although some of them can be purified from natural sources [10, 11]. Chemical approaches usually require very toxic reagents and display low selectivity, which involves multiple protection and deprotection steps [12]. On the contrary, enzymes are considered very selective and environmentally friendly biocatalysts. In this sense, some retaining glycosidases are notable for using cheap substrates to catalyze the synthesis of glycoconjugates by transglycosylation [13, 14]. By this reaction, a sugar unit, passing through a glycosyl-enzyme intermediate, is transferred from a donor to an acceptor compound other than water. For xylosides, the reaction is known as transxylosylation and it can be catalyzed by both retaining β-xylosidases and endoxylanases. β-xylosidases have an exo-mode of action, and they form well-defined products transferring exclusively xylose residues. However, to do so, they need the disaccharide xylobiose or short XOS as donors [15], which are expensive carbohydrates obtained from xylan or by chemical synthesis. In a previous work [16] we proposed a novel, green alternative, for the synthesis of DHNX, based on transxylosylation. However, a quite high concentration of xylobiose showed to be necessary to achieve maximal yields, and the high cost of this substrate was pointed out as a major issue of this approach. On the contrary, certain endoxylanases are able to use xylan itself as donor, allowing a more cost-effective production, although in this case the sugars transferred are not only single xylose units, but mainly XOS of variable length, which necessarily leads to produce a mixture of xylosides and reduces the industrial interest of the process [10].

Hence, an enzyme cascade combining both biocatalysts may be the way to overcome these drawbacks and exploit strengths. The approach consists on using xylan as raw material to be converted into XOS by an endoxylanase, preferably one unable to catalyze the direct transglycosylation of the selected acceptor. As these oligosaccharides are released, they can serve as transxylosylation donors in a reaction catalyzed by a β-xylosidase, which transfers xylose units from the non-reducing end of the XOS to the selected acceptor. Therefore, this cascade could constitute an effective one-pot strategy to synthetize value-added xylosides at a more competitive cost, valorizing the xylans derived from biomass.

**Fig. 1:**
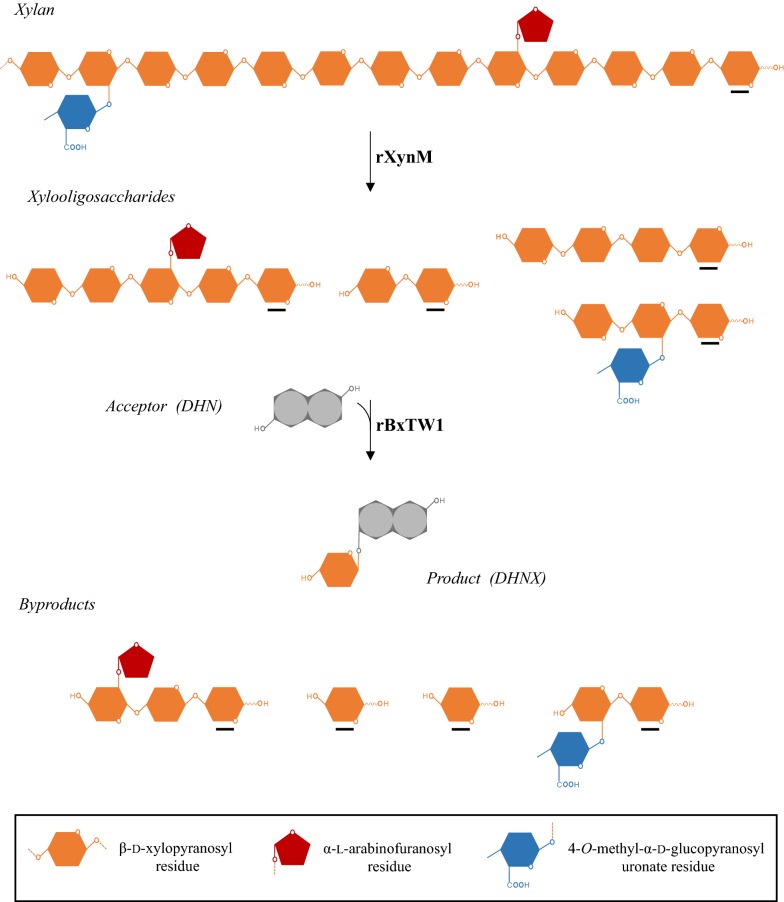
Enzymatic cascade for one-pot synthesis of DHNX from xylan and DHN. The endoxylanase rXynM hydrolyzes the polysaccharide releasing a mixture of XOS. The β-xylosidase rBxTW1 transfers xylopyranose units from the non-reducing end of the XOS to DHN by transglycosylation, forming DHNX. By this approach, XOS can be consumed up to the reducing-end residue or a branching point (byproducts). Reducing ends are underlined in black

In this work, we validated this approach, producing the recombinant endoxylanase XynM from the ascomycete *T. amestolkiae* in the yeast *P. pastoris* and combining this biocatalyst with the recombinant β-xylosidase rBxTW1 from the same fungus, which had previously been characterized in our group [16]. Through this enzyme cascade, DHNX was produced from beechwood or birchwood xylan and 2,6-dihydroxynaphthalene (2,6-DHN) (Fig. 1). The optimum conditions for the process were determined by a Central Composite Design (CCD). To the best of our knowledge, this is the first report on the application of an enzyme cascade to exploit xylan as a transglycosylation donor to produce a compound with pharmacological interest.

## Materials and methods

### Materials

Xylans from beechwood, birchwood and oat spelt, *p*-nitrophenyl β-d-xylopyranoside (*p*NPX) and 2,6-DHN were purchased from Sigma-Aldrich. Larchwood xylan was from Koch-Light laboratories. Xylans from wheat straw, barley straw and corn stover were kindly provided by Laboratorios Andrómaco. SuperScript Reverse Transcriptase II Kit, Pierce BCA Protein Assay Kit, *Pichia* Expression Kit including pPIC9 vector and EnzChek^®^ Ultra Xylanase Assay Kit were purchased from Thermo Fisher Scientific. RNeasy Plant Mini Kit was from Qiagen.

### Enzyme and protein assays

Endoxylanase activity was measured by the release of reducing sugars according to the Somogyi-Nelson method [17]. Beechwood xylan was selected as substrate and one unit of endoxylanase activity was defined as the amount of enzyme necessary for releasing 1 μmol of reducing sugars per minute. The standard reaction was performed as described elsewhere [18].

β-Xylosidase activity was measured spectrophotometrically by the release of *p*-nitrophenol (*p*NP) (ε_410_ = 15,200 M^−1^ cm^−1^) from *p*-nitrophenyl β-d-xylopyranoside. One unit of β-xylosidase activity was defined as the amount of enzyme that hydrolyzes 1 μmol of *p*NPX per min in standard conditions. The standard reaction was carried out according to the protocol described by Nieto-Domínguez et al. [15], but replacing the citrate sodium buffer with 50 mM sodium acetate buffer (pH 5).

Protein quantification was carried out by the bicinchoninic acid method, selecting bovine serum albumin as the standard and using the Pierce™ Protein Assay Kit according to the manufacturer’s instructions.

### Cloning of the endoxylanase gene and activity screening

The *T. amestolkiae* CIB strain is deposited in the IJFM (Instituto “Jaime Ferrán” de Microbiología) culture collection of the Centro de Investigaciones Biológicas, CSIC (Madrid, Spain) with the reference A795.

Fungal inoculum and culture were performed as previously described [19] and 2% beechwood xylan was added as carbon source and endoxylanase inducer. Fungal biomass was collected at day 3 post-inoculation when the endoxylanase activity was maximal [18]. RNA was isolated from *T. amestolkiae* by using the RNeasy Plant Mini Kit in accordance with the manufacturer’s instructions. An RT-PCR was carried out from the isolated RNA by using the SuperScript Reverse Transcriptase II Kit following the manufacturer’s instructions (the reaction mix contained 25 ng/μL purified fungal RNA and 25 ng/μL Oligo(dT)_12–18_ primers). The cDNA obtained in this way was used as template for the amplification of *xynm* gene coding for XynM from *T. amestolkiae* (GenBank accession no. KX641268). Primers were designed excluding the signal peptide, which was predicted by the SignalP 4.1 server (http://www.cbs.dtu.dk/services/SignalP/). The restriction sites for *Eco*RI and *Not*I were respectively added to the 5′ and 3′ primers, together with three extra nucleotides in order to increase the efficiency of the endonucleases. Primer sequences were as follows: XynM Fw, 5′-TCTGAATTCTCGACTCCTATCAACTACGTTCAGA-3′ and XynM Rv, 5′-ACTGCGGCCGCCTAAGAGCCAGACACGGTCACG-3′. PCR reaction mixtures contained 8 μL cDNA, 1 μL PCR buffer, 1.5 mM MgCl_2_, 0.8 mM deoxynucleoside triphosphates, 0.5 μM each primer, and 1 U of Expand™ High Fidelity PCR System DNA polymerase (Sigma-Aldrich) in a final volume of 50 μL. The reaction mixtures were denatured at 94 °C for 5 min and then subjected to 34 cycles of amplification, each at 94 °C for 30 s, 60 °C for 30 s, and 72 °C for 1 min, followed by a final extension step at 72 °C for 7 min. Control reactions lacking DNA template were simultaneously performed.

The PCR product encoding the mature XynM was ligated into the pPIC9 vector. This construction keeps the gene under the control of the methanol-induced promoter AOX1 and fused with the α-factor signal sequence. This construction was linearized with *Sac*I (New England Biolabs) and used for transforming *P. pastoris* GS115 and KM71. Transformed colonies were cultured on Yeast Nitrogen Base plates in the absence of histidine as selection marker (YNB–His).

Selected clones from YNB–His were cultured on YPM medium plates [1% yeast extract, 1% peptone, 1% g/L agar and 1.5% methanol (v/v)], in order to identify positive transformants. The yeasts were cultured for 24 h at 28 °C. The plates were covered with a solution of 50 μM 6,8-difluoro-4-methylumbelliferyl β-d-xylobioside (Component A of EnzChek^®^ Ultra Xylanase Assay Kit) in 50 mM sodium acetate buffer and 0.8% agar. After solidifying, the plate was incubated at 50 °C for 20 min. Clones with endoxylanase activity hydrolyze 6,8-difluoro-4-methylumbelliferyl β-d-xylobioside, releasing the fluorescent 6,8-difluoro-4-methylumbelliferone, which is visualized under UV light using the Gel Doc XR+ system (Bio-Rad).

### Production and purification of rXynM and rBxTW1

*Pichia pastoris* positive clones for endoxylanase production were cultured in triplicate in order to identify the one displaying the maximal secretion of endoxylanase activity. Cultures were performed as previously described [16] and samples were withdrawn daily in order to determine endoxylanase activity and estimate yeast growth measuring absorbance at 600 nm (A_600_).

7-day-old cultures of the GS115 selected clone were harvested and centrifuged at 10,000*g* and 4 °C for 20 min. Then, supernatants were filtered through 0.8-, 0.45-, and 0.22-μm disc filters (Merck-Millipore) and the crude was concentrated and dialyzed against 10 mM sodium phosphate buffer (pH 6) by ultrafiltration, using a 5-kDa cutoff membrane.

rXynM was purified by a single step of fast protein liquid chromatography using an ÄKTA Purifier system (GE Healthcare). The dialyzed enzyme crude was loaded onto a 5-mL HiTrap QFF cartridge (GE Healthcare), equilibrated in 10 mM sodium phosphate buffer (pH 6) and a flow rate of 1 mL/min was kept during the entire process. Elution was carried out by applying a linear gradient of 1 M NaCl from 0 to 30% for 35 min. Then the column was washed with 1 M NaCl for 10 min and re-equilibrated with the starting buffer for 10 min. Absorbance at 280 nm was monitored and fractions corresponding to the peak of maximal endoxylanase activity were collected and pooled together to be dialyzed and concentrated by ultrafiltration using 5-kDa cutoff Amicon Ultra-15 centrifugal devices (Merck-Millipore). The purified protein was stored at 4 °C.

The recombinant BxTW1 produced in *P. pastoris* was obtained and purified as previously reported [16].

### Characterization of rXynM

The estimated molecular mass of the enzyme and its purity were evaluated by SDS-PAGE in 12% acrylamide gels [20] using Precision Plus Protein TM Dual Color Standards (Bio-Rad) and staining with Coomassie Brilliant Blue R-250. The accurate protein molecular mass was determined by MALDI-TOF in an Autoflex III (Bruker Daltonics).

The determination of optimum pH and temperature together with thermal and pH stabilities for 72 h were performed as described elsewhere [15].

Enzyme kinetics were determined against beechwood xylan, measuring endoxylanase activity with increasing concentrations of substrate from 1 to 45 g/L and adjusting the experimental data by least-squares to the Lineweaver–Burk linear equation of the Michaelis–Menten model.

### Development of enzyme cascades

A preliminary assay combining rXynM and rBxTW1 in order to obtain DHNX was carried out to check the feasibility of the approach. The reaction mixture contained 40 g/L beechwood xylan, 3 g/L 2,6-DHN, 50 mM sodium acetate buffer (pH 5), 2.1 μM rXynM, 3.0 μM rBxTW1 and was incubated at 45 °C and 1200 rpm for 120 min. The reaction was stopped by heating at 100 °C for 5 min. The production of the expected xyloside was analyzed by HPLC in an Agilent 1200 series instrument equipped with a ZORBAX Eclipse XDB-C18 column (Agilent). The system was equilibrated in acetonitrile/H_2_O 10:90 v/v (both containing 0.1% acetic acid) and the reaction products were eluted in a linear gradient from 10 to 20% acetonitrile as reported by Nieto-Domínguez et al. [16]. Absorbance was monitored at 220 nm and the concentration of the product was determined from a calibration curve of 2,6-DHN.

CCD was selected as a suitable response surface method for optimizing the reaction conditions in order to achieve the maximal production of DHNX combining rBxTW1 and rXynM. Two different experimental matrices were generated using the Design-Expert^®^ software version 10.0.1.0 (Stat-Ease Inc.) for beechwood and birchwood xylans (Additional file 1: Tables S1 and S3). Temperature, reaction time, concentration of endoxylanase and β-xylosidase, and pH, were selected as independent variables. These parameters were studied at three levels: low, middle and high, whereas the amount of obtained product was defined as response. The values of low and high levels were established by a one factor at a time approach (data not shown). 2,6-DHN was added at initial concentration of 3 g/L. The concentration of the polysaccharide was included in CCD only for birchwood xylan, while beechwood xylan was added to an initial concentration of 80 g/L, as will be explained later (see “Enzyme cascade for the synthesis of 2-(6-hydroxynaphthyl) β-d-xylopyranoside from beechwood xylan” section). The response data obtained for each condition were analyzed using the Design-Expert^®^ software, in order to describe the enzyme cascade as a multiparametric equation. The reaction model allowed the prediction of the specific conditions for the maximum production of the xyloside with each polysaccharide.

The peak of the xyloside was identified on the basis of the retention time of the same product obtained according to the enzymatic method described elsewhere [16]. The product peak obtained in the conditions that gave the maximum yields of synthesis was collected, and its identity was confirmed by mass spectrometry. The DHNX derived from beechwood was analyzed by NMR to verify the structure and evaluate purity.

The optimal conditions determined for beechwood xylan were applied to other xylans from different plant sources, which were assayed at a concentration close to their limit of solubility. The selected substrates and concentrations were as follows: 60 g/L birchwood xylan, 80 g/L larchwood xylan, 80 g/L oat spelt xylan, 50 g/L wheat straw xylan, 80 g/L barley straw xylan, 80 g/L corn stover xylan.

### Mass spectrometry

Product samples were analyzed by direct infusion on a Bruker HCT Ultra ion trap. Ionization was performed by electrospray with methanol as ionizing phase in the negative reflector mode.

### Gas chromatography

The monosaccharide composition of the assayed xylans was determined by gas chromatography on a 7890A instrument (Agilent). Samples of each polysaccharide were hydrolyzed with 3 M trifluoroacetic acid (TFA) at 121 °C for 1 h. Then, they were derivatized and analyzed as reported by Bernabé et al. [21]. The monosaccharide content was quantified using inositol as the internal standard.

### NMR

The identification and assessment of purity of DHNX were performed by NMR. Samples were prepared in 500 µL of deuterated water (D_2_O). Experiments were acquired at 298 K, using a Bruker AVANCE 600 MHz spectrometer equipped with a cryogenic probe. 1D ^1^H NMR spectra and ^1^H-^13^C HSQC experiments were acquired to assign all NMR signals. Concentration was determined by comparison of the integrals of the signals in the 1D spectra. Diffusion Ordered Spectroscopy (DOSY) was performed using the standard Bruker pulse sequence (ledbpgp2s), acquiring 16 gradient points, with 64 scans each, between 2 and 95% gradient intensity using a diffusion time delay of 0.3 s and 3000 µs wide pulse gradient. The size of polysaccharides was estimated by comparing the obtained diffusion coefficients with a calibration curve performed with oligo- and polysaccharides of known molecular mass [22, 23].

## Results and discussion

### Enzyme production and characterization

**Fig. 2:**
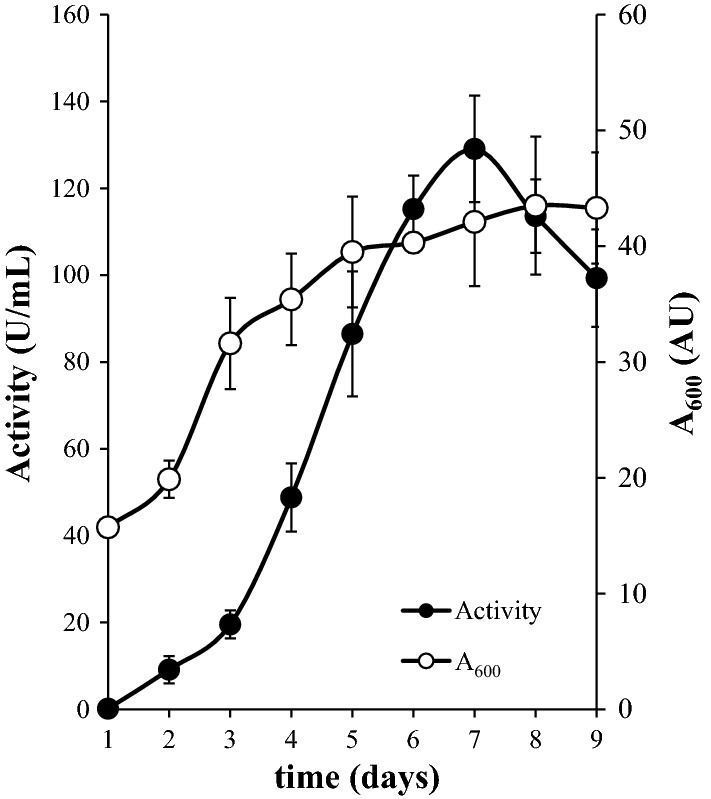
Extracellular endoxylanase activity and absorbance at 600 nm across time of the selected clone of *P. pastoris* cultured in YEPS medium

The mature gene *xynm*, without introns and native peptide signal, was successfully expressed in *P. pastoris*. The screening in YPM medium plates using 6,8-difluoro-4-methylumbelliferyl β-d-xylobioside revealed several clones displaying high fluorescence, which were cultured in liquid medium in order to identify the highest producer of endoxylanase activity. The clones showed a similar behavior, achieving the highest endoxylanase activity after 7 incubation days. One clone from strain GS115 displayed a slightly higher maximal value (129 U/mL), and was selected for enzyme production. Figure 2 displays the profile of secreted activity together with the A_600_, as an estimation of the yeast growth.

rXynM was purified by FPLC from 7-day-old liquid cultures through a single anion-exchange chromatography step. The purification process recovered 33% of the total loaded activity, and the specific activity increased from 15.9 U/mg in the protein crudes to 122.0 U/mg in the collected fractions, which corresponds to a degree of purification of 7.7. This value, and the initial production titer of 1.1 g/L are values close to those reported for other fungal xylanases heterologously produced in *P. pastoris* [24, 25].

Both production and purification of rXynM represented a remarkable enhancement in comparison to the native enzyme. The fungus reached an overall xylanase activity (considering together endoxylanases, β-xylosidases and auxiliary enzymes) of 85 U/mL, a value below that determined in the yeast, despite it secretes rXynM as the sole xylanolytic enzyme [18, 19]. The purification of the native XynM required two steps of FPLC and allowed recovering 5.3% of the initial activity, whereas rXynM was purified in a single chromatographic step (Additional file 1: Figure S1) and the recovered activity was of 32.9%, sixfold higher to that of the native protein [18]. Thus, it can be concluded that *P. pastoris* and the pPIC9 system were suitable choices for the recombinant production of XynM.

**Fig. 3:**
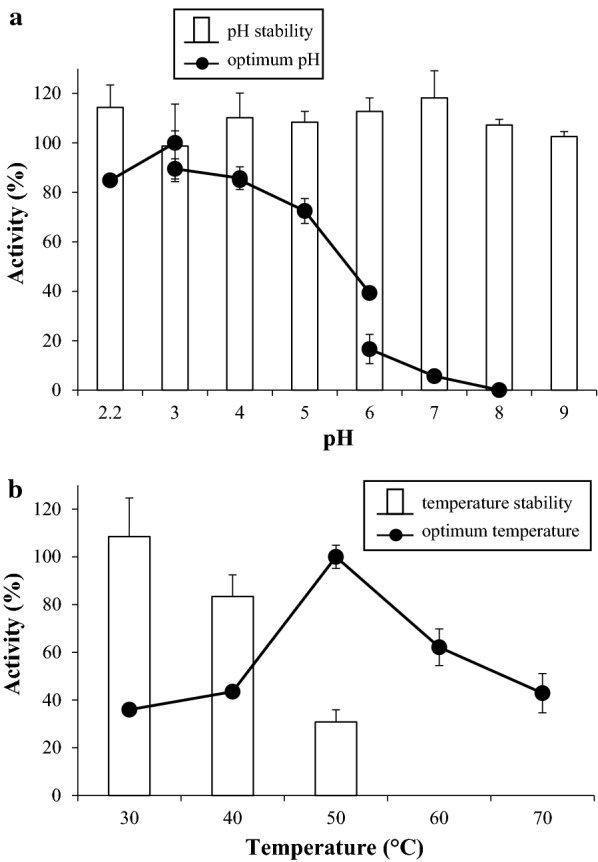
pH (**a**) and temperature (**b**) profiles of rXynM. **a** The line indicates the effect of pH on enzyme activity, and the bars show its stability over a range of pH values from 2.2 to 9 after 72 h. **b** The line indicates the effect of temperature on enzyme activity, and the bars show its stability over a range of temperatures from 30 to 50 °C after 72 h

The biochemical analysis of rXynM indicated properties very close to the ones reported for the native enzyme [18]. The molar mass determined by MALDI-TOF MS was 19,968 Da (Additional file 2), the optimum pH of the recombinant protein ranged between 3 and 4, and the optimal temperature was 50 °C. Concerning its stability, rXynM tolerates the wide range of pH assayed (2.2–9.0) and temperature values up to 40 °C, while it appeared to be rapidly denatured at 50 °C (Fig. 3).

The same similarities were observed for the kinetic parameters. The calculated values for *K*_*m*_ and *V*_*max*_ were 6.79 ± 1.7 g/L and 125.2 ± 11.1 U/mg respectively, which are very close to the 5.5 g/L and 129 U/mg reported for the native endoxylanase [18].

### Enzyme cascade for the synthesis of 2-(6-hydroxynaphthyl) β-d-xylopyranoside from beechwood xylan

In a previous work, we described the feasibility of rBxTW1 for synthesizing the selective antiproliferative DHNX using xylobiose and 2,6-DHN as transxylosylation donor and acceptor, respectively [16]. However, the high cost of the disaccharide represents a major drawback for the industrial potential of the process. As mentioned before, combining an endoxylanase and a β-xylosidase in a cascade reaction may lead to the exploitation of xylan as a cheap source of sugar donors. The main hindrance for the application of an enzyme cascade is the requirement of establishing a set of valid conditions for different biocatalysts. The joint use of the glycosidases rXynM and rBxTW1 produced by the ascomycete *T. amestolkiae* overcomes this limitation, because they have similar properties, achieving their maximal activity under acidic and mesophilic conditions. In addition, the endoxylanase had previously shown its ability to hydrolyze xylan with considerably good yields, releasing mainly neutral linear XOS from 2 to 4 units (and negligible quantities of xylose), which can be efficiently used by BxTW1 [15, 18]. It should be noted that, to date, rBxTW1 is the only biocatalyst reported to be able to catalyze the transxylosylation of the bulky DHN, therefore a direct transfer of XOS from xylan to this acceptor by XynM was not expected.

**Fig. 4:**
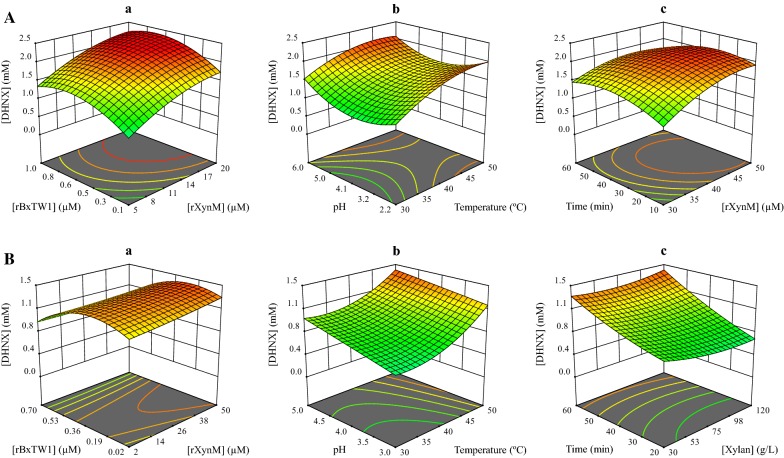
3D representations of the calculated models for the production of DHNX from beechwood (**A**) and birchwood (**B**) xylans. The 3D graphs were generated by plotting the selected pairs of parameters as X1 and X2 while the remaining ones were kept at their optimal values. Selected X1 and X2 parameters are, respectively, concentration of rXynM and rBxTW1 (**A**-a, **B**-a); temperature and pH (**A**-b, **B**-b); concentration of rXynM and time (**A**-c); concentration of xylan and time (**B**-c). The production of DHNX was selected as response in every case

The first attempt to apply the cascade in the conditions of the preliminary assay (see “Development of enzyme cascades” section) led to the successful synthesis of the desired xyloside, but in a low concentration (0.12 mM). With the purpose of optimizing the production yield, the effect of several reaction parameters was analyzed by CCD, one of the response surface methods with highest predictability [26]. The concentrations of xylan (source of donors) and 2,6-DHN (acceptor), although being essential for the process, were fixed close to their solubility limits since the production levels were directly proportional to the selected concentrations (data not shown). From the analysis of the experimental data, a quadratic polynomial equation was generated as a model of the enzyme cascade (Additional file 1: Table S1 and Eq. S1). Figure 4A illustrates the influence of the studied parameters in the production of DHNX.

This equation considered the production of DHNX as a function of reaction time, temperature, pH and the concentrations of rXynM and rBxTW1. Chromatograms did not show significant quantities of any potential xyloside of DHN containing more than one xylose unit, suggesting that, as expected, rXynM was not able to catalyze direct transxylosylation from xylan in the tested conditions. An analysis of variance (ANOVA) was carried out by the Design-Expert^®^ software, validating that the obtained model matched the experimental data (Additional file 1: Table S2). Once verified, the polynomial equation was applied to predict the conditions for maximum xyloside production, which were further assayed experimentally. The highest concentration of DHNX was expected to be reached at 3 g/L 2,6-DHN, 80 g/L beechwood xylan, 12.5 μM rXynM, 0.7 μM rBxTW1, pH 6, 45.7 °C and 32 min as reaction time. Under these conditions, the model predicted 2.11 mM product and the experimental value was 2.02 mM (0.59 g/L), which in terms of converted acceptor indicated a 10.7% yield. The identity of the product was confirmed by ESI–MS (Additional file 2) and NMR (Additional file 1: Table S5 and Figure S2), besides by its retention time. By this way, the prediction capacity of the model was demonstrated and the production of 2-(6-hydroxynaphthyl) β-d-xylopyranoside increased 17-fold, from 0.12 mM in the conditions of the preliminary assay to 2.02 mM after optimization. In addition, NMR analyses indicated the presence of a certain amount of xylan or XOS in the sample (Additional file 1: Figure S3). The comparison of the integrals of the separate signals, allowed deducing that the proportion between DHNX and the remaining xylose units (from the residual xylan) was 4.2–4.8, indicating a purity around 89–90% (g/g).

The maximum production achieved with the previous enzymatic method, with rBxTW1 as the only catalyst, was 1.6 mM (0.47 g/L, 8.5% acceptor conversion), which is lower than the one attained for the enzyme cascade. This increased yield may be explained by the synergism between the xylanases selected. The β-xylosidase consumes the XOS released by the endoxylanase, avoiding its inhibition by accumulation of the oligosaccharides produced. More importantly, rXynM release not only xylobiose, but also XOS with higher degree of polymerization [18]. This circumstance is relevant because the xylosidase acts on XOS from the non-reducing end by an *exo*-mechanism of transxylosylation, preventing the release of free xylose at the reducing end. Instead, this enzyme produces shorter XOS that can be reused as donors, reducing the quantity of monomeric xylose released and delaying the product inhibition of rBxTW1.

The above data confirm that the enzyme cascade evaluated is a suitable approach for replacing xylobiose by xylan as sugar donor for the synthesis of DHNX and, additionally, show that this strategy improves the production of this compound. To the best of our knowledge, this is the first report of a successful approach for xylan exploitation to produce a glycoconjugate with pharmacological interest by means of an enzyme cascade.

### Exploitation of xylans from different plant sources

The use of beechwood xylan at the laboratory level is very frequent due to its commercial availability and high xylose content (≥ 90% according to the manufacturer), which indicates a low grade of substitution. However, the structure of xylan, like that of most hemicelluloses, is known to vary depending on the plant source [2]. The most important changes reported occur at the side chains level, with differences in size, frequency and nature of the branches. If the conditions determined to synthetize DHNX from beechwood xylan are applied to other xylans, their structural differences may affect the number of target sites for rXynM in the main backbone, and therefore also the yield of the enzymatic production of DHNX. Several xylans from different plant sources were tested as xylose providers to assess the actual influence of their structural differences on the DHNX yields, using the same enzyme cascade and optimal conditions previously set up for beechwood xylan. Among the chosen substrates, oat spelt and birchwood are highly referred [1, 3, 27], larches are one of most common softwoods in boreal, montane and subalpine forests [28], and wheat straw, barley straw and corn stover are among the most abundant biomass residues in global agriculture [29].

Table 1 displays the yields of DHNX from all xylans tested. As compared with beechwood xylan, the production is reduced more than tenfold in most cases. These results strongly suggest that the optimal conditions determined by CCD for beechwood xylan cannot be extended to xylans from other sources. The analysis of the monosaccharide composition of each polysaccharide, which was very helpful to understand the low yields obtained, is also displayed in the table together with the substrate concentration in the reaction.

**Table 1:** Yield of the 2-(6-hydroxynaphthyl) β-d-xylopyranoside produced using different xylans as sugar donors and concentration and monosaccharide composition of the xylans

Source	Yield (%)	Xylan (g/L)	Composition (%)			
			Xylose	Arabinose	Galactose	Glucose
Beechwood	10.7	80	100.0	–	–	–
Barley straw	0.63	80	90.6	9.4	–	–
Birchwood	1.07	60	100.0	–	–	–
Corn stover	0.52	80	91.0	6.6	–	2.4
Larchwood	1.15	80	70.1	10.7	1.4	17.8
Oat spelt	1.90	80	82.8	9.4	–	7.7
Wheat straw	0.19	50	90.9	9.1	–	–

**Fig. 5:**
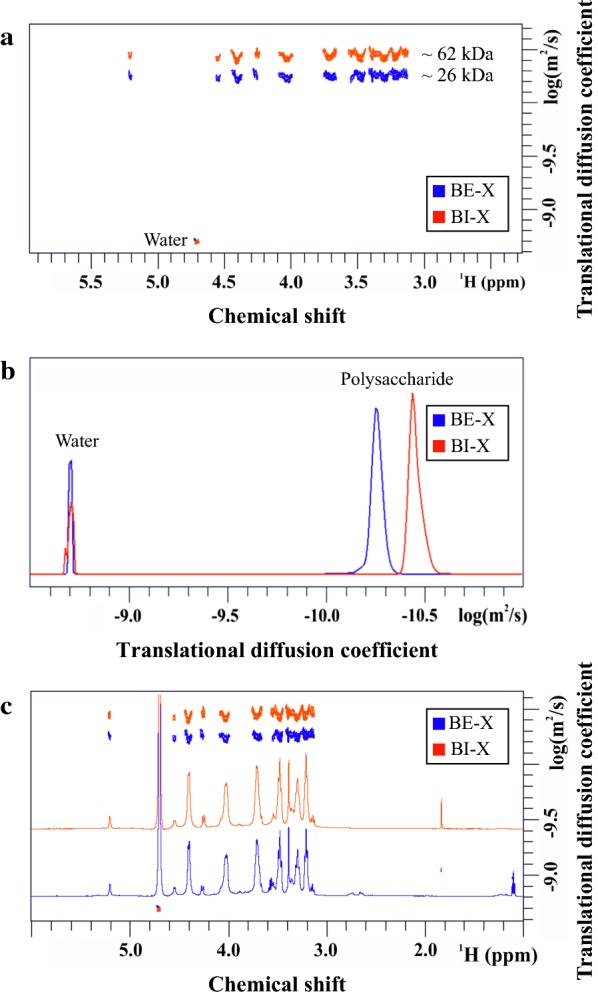
DOSY NMR analyses. Signals corresponding to water were used as reference to ease the comparison between both polysaccharides. **a** Superimposition of DOSY spectra from beechwood xylan (BE-X) in blue and birchwood xylan (BI-X) in red. The x-axis represents chemical shift and the y-axis shows the translational diffusion coefficient. Labels indicate the signals corresponding to the solvent (water) and the weight average of the polysaccharides. **b** Projection of molecular size distribution of BE-X (blue) and BI-X (red). The x-axis shows the differences at the molecular weight level in terms of translational diffusion coefficient. Labels indicate the peaks corresponding to the polysaccharides and the solvent (water). **c** Superimposition of the DOSY and ^1^H 1D spectra of BE-X (blue) and the BI-X (red). Differences in the molecular weight are observed (different diffusion coefficient). Moreover, no acetylation pattern around 2 ppm is observed for both xylans. The peaks at 1.85 ppm/− 9.03 LogD in BI-X and at 1.10 ppm/− 8.98 LogD in BE-X correspond to low molecular weight impurities. The x-axis represents chemical shift and the y-axis shows the translational diffusion coefficient

All the tested xylans have high xylose content, but outstanding differences can be noticed. Barley straw and corn stover showed around 10% of monosaccharides other than xylose, mainly arabinose, and this amount increased up to ~ 18% in the case of oat spelt and ~ 30% for larchwood, that contains more glucose than arabinose. On the contrary, beechwood and birchwood xylans are formed almost exclusively by xylose. The presence of carbohydrates apart from xylopyranose does not allow determining the accurate frequency and size of the side chains, but it does confirm the presence of branches, which may hinder the action of both rXynM and rBxTW1 and could explain the different yields found. However, the case of birchwood requires a different explanation since, as beechwood xylan, it is basically composed of xylose. Therefore, despite the big difference of yield observed, it may be assumed that both are almost linear polysaccharides (without considering acetylations which would not be detected by gas chromatography). Interestingly, birchwood xylan showed lower solubility than beechwood, suggesting some structural difference between them. The analysis of both xylans by DOSY NMR [22, 23] (Fig. 5) revealed that the two xylans have different molecular size distributions, being the weight average remarkably higher for birchwood (~ 62 kDa) than for beechwood xylan (~ 26 kDa). In addition, the ^1^H spectra of both polysaccharides did not show detectable signals corresponding to acetylations (Fig. 5c), confirming weight average as the only relevant difference between them. This circumstance may have a deep influence on the observed yield, since the optimal quantity of endoxylanase for beechwood xylan is probably not enough for birchwood. This biocatalyst is crucial to the process, as the internal breakage of the polysaccharide chain exposes xylose units at non-reducing ends, thus increasing the number of available donors for rBxTW1. It should be noticed that an insufficient donor concentration can lead the β-xylosidase to consume quickly any previously formed xyloside by the competing hydrolytic reaction. Taking this into account, a correction in the rBxTW1/rXynM balance that shifts toward the endoxylanase might substantially increase the production of DHNX. An additional multiparametric model was developed with the aim of validating this hypothesis and assessing the relevance of molecular size of the starting polysaccharide substrate in the enzymatic exploitation of xylan.

### Enzyme cascade for the synthesis of 2-(6-hydroxynaphthyl) β-d-xylopyranoside from birchwood xylan

As for beechwood xylan, the reaction model was developed by CCD, although the concentration of polysaccharide was included as a variable of the model in this case, since the one factor at a time experiments suggest that the relation between amount of polysaccharide and yield was not entirely linear. The analysis of the response values determined for each experimental condition led to obtain a new quadratic polynomial equation (Additional file 1: Table S3 and Eq. S2). Figure 4B illustrates the influence of the studied parameters in the production of DHNX.

The model was validated by ANOVA analysis (Additional file 1: Table S4) and the conditions of maximal production were calculated. The xyloside was predicted to reach 1.4 mM (0.41 g/L) in a reaction containing 3 g/L 2,6-DHN, 60 g/L birchwood xylan, 50 μM rXynM, 0.24 μM rBxTW1 at pH 5 and 50 °C for 120 min. The value determined experimentally was 1.3 mM (0.38 g/L) confirming the accuracy of the equation. The retention time in HPLC and the ESI-MS analysis (Additional file 2) confirmed the identity of the product.

A sevenfold enhancement of the DHNX production was achieved using the specific conditions optimized for birchwood xylan instead of those set up for beechwood. Despite this fact, the production observed (7.0%) is still somewhat below the conversion for beechwood xylan (Table 1). The greatest difference between both models was the value of the endoxylanase/β-xylosidase (mole/mole) balance required for maximal production. For beechwood xylan it is 18:1, but it changes to 125:1 in the case of birchwood xylan. Such an increase in the ratio was correlated with the higher molecular size of the last xylan, highlighting that, in addition to the branching degree, this parameter must be carefully considered to optimize the enzymatic exploitation of any selected xylan.

Taking these data into account, it has been proven that experimental drawbacks caused by the different nature of the substrates can be overcome by the development of a specific model. On the other side, this work has focused on virtually non-branched xylan, but in nature the main chain of this hemicellulose is frequently substituted, mainly by arabinose and α-d-glucuronic acid. In fact, 4-*O*-methyl-d-glucuronoxylans and arabinoxylans are the predominant hemicelluloses in hardwoods and cereals, respectively [2]. The presence of these side chains may challenge an enzyme cascade like the one reported in this work, by hindering the activity of both rXynM and rBxTW1. Therefore, the next step on the exploitation of xylan should focus on more complex but also more abundant classes of xylan. Future research may study the addition of suitable debranching biocatalysts to the cascade, as α-l-arabinofuranosidases for arabinoxylans or α-glucuronidases for glucuronoxylan. Additionally, it is necessary to consider the potential of alternative endoxylanases, as the ones from the GH10 family, which are more efficient than GH11 enzymes on substituted backbones of xylan [30]. In this sense, *T. amestolkiae* represents also an interesting source for both GH10 and auxiliary xylanolytic biocatalysts [19].

## Conclusions

An enzyme cascade for synthesizing the antiproliferative 2-(6-hydroxynaphthyl) β-d-xylopyranoside from beechwood and birchwood xylans was developed, allowing the replacement of the high cost xylobiose by xylan, easily obtained from plant biomass. Optimization of reaction conditions by response surface methods led to a remarkable increase of production, overcoming the yield achieved using the disaccharide in the case of beechwood. The analyses performed demonstrate the relevance of the molecular size of the polysaccharide, suggesting that specific modeling is required depending on the xylan source. This work highlights the combination of fungal xylanolytic enzymes as an approach for obtaining value-added products from biomass.

## Supplementary information

**Additional file 1: Figure S1.** (A) SDS-PAGE analysis of isolated rXynM. (B) Accurate determination of the molecular mass of rXynM by MALDI-TOF. **Table S1.** Central composite experimental design for optimizing the synthesis of 2-(6-hydroxynaphthyl)-β-d-xylopyranoside from beechwood xylan. **Table S2**. ANOVA results for the multiparametric model using beechwood xylan. **Equation S1.** Quadratic model equation for the production of DHNX from beechwood xylan. **Table S3.** Central composite experimental design for optimizing the synthesis of 2-(6-hydroxynaphthyl)-β-d-xylopyranoside from birchwood xylan. **Table S4**. ANOVA results for the multiparametric model using birchwood xylan. **Equation S2.** Quadratic model equation for the production of DHNX from birchwood xylan. **Table S5.** Chemical shift data from 2-(6-hydroxynaphthyl) β-d-xylopyranoside. **Figure S2.** ^1^H-^13^C HSQC edited spectra (blue, CH/CH_3_; red, CH_2_) and ^1^H 1D spectra (orange) of DHNX obtained from beechwood xylan. **Figure S3.** Superimposition of the ^1^H 1D spectra of the beechwood xylan substrate (blue) and the DHNX (red). The raw beechwood background is observed in the DHNX sample. The ratio of integrals of the non-overlapping peaks highlighted was employed to estimate the purity..

**Additional file 2.** Mass spectrometry. Mass spectra (negative mode) of the isolated DHNX from beechwood and birchwood are displayed below. The product adducts identified are appropriately labeled.

## Data Availability

All relevant data have been included in this published article and its additional files.

## Abbreviations

A_600_: absorbance at 600 nm
ANOVA: analysis of variance
BE-X: beechwood xylan
BI-X: birchwood xylan
CCD: central composite design
2,6-DHN: 2,6-dihydroxynaphthalene
DHNX: 2-(6-hydroxynaphthyl) β-d-xylopyranoside
D_2_O: deuterated water
DOSY: diffusion ordered spectroscopy
ESI–MS: electrospray ionization–mass spectrometry
FPLC: fast protein liquid chromatography
MALDI-TOF: matrix-assisted laser desorption ionization-time of flight mass spectrometry
NMR: nuclear magnetic resonance
*p*NP: *p*-nitrophenol
*p*NPX: *p*-nitrophenyl β-d-xylopyranoside
rBxTW1: recombinant BxTW1
rXynM: recombinant XynM
TFA: trifluoroacetic acid
XOS: xylooligosaccharides
YNB: yeast nitrogen base
YPM: yeast extract peptone methanol
