# Supplementary material for: Exploiting xylan as sugar donor for the synthesis of an antiproliferative xyloside using an enzyme cascade
Source: Microb Cell Fact. 2019 Oct 10;18:174. doi: 10.1186/s12934-019-1223-9 (PMC6788083; doi:10.1186/s12934-019-1223-9)
Supplement: Supplementary file 1 — Additional file 1: Figure S1. (A) SDS-PAGE analysis of isolated rXynM. (B) Accurate determination of the molecular mass of rXynM by MALDI-TOF. Table S1. Central composite experimental design for optimizing the synthesis of 2-(6-hydroxynaphthyl)-β-d-xylopyranoside from beechwood xylan. Table S2. ANOVA results for the multiparametric model using beechwood xylan. Equation S1. Quadratic model equation for the production of DHNX from beechwood xylan. Table S3. Central composite experimental design for optimizing the synthesis of 2-(6-hydroxynaphthyl)-β-d-xylopyranoside from birchwood xylan. Table S4. ANOVA results for the multiparametric model using birchwood xylan. Equation S2. Quadratic model equation for the production of DHNX from birchwood xylan. Table S5. Chemical shift data from 2-(6-hydroxynaphthyl) β-d-xylopyranoside. Figure S2. 1H-13C HSQC edited spectra (blue, CH/CH3; red, CH2) and 1H 1D spectra (orange) of DHNX obtained from beechwood xylan. Figure S3. Superimposition of the 1H 1D spectra of the beechwood xylan substrate (blue) and the DHNX (red). The raw beechwood background is observed in the DHNX sample. The ratio of integrals of the non-overlapping peaks highlighted was employed to estimate the purity.. [file 12934_2019_1223_MOESM1_ESM.pdf]

ADDITIONAL FILE 1

AN ENZYME CASCADE FOR 2-(6-HYDROXYNAPHTHYL) β-D-XYLOPYRANOSIDE SYNTHESIS FROM XYLAN

Manuel Nieto-Domínguez[a], José Alberto Martínez-Fernández[a], Beatriz Fernández de Toro[b], Juan A. Méndez-Líter[a], Francisco Javier Cañada[b], Alicia Prieto[a], Laura I. de Eugenio[a]* and María Jesús Martínez[a]*

[a] Biotechnology for Lignocellulosic Biomass Group and [b] NMR and Molecular Recognition Group, Centro de Investigaciones Biológicas (CIB-CSIC), c/Ramiro de Maeztu 9, 28040, Madrid, Spain.

*Correspondence: lidem@cib.csic.es (L.I. de Eugenio); mjmartinez@cib.csic.es (M.J. Martínez).

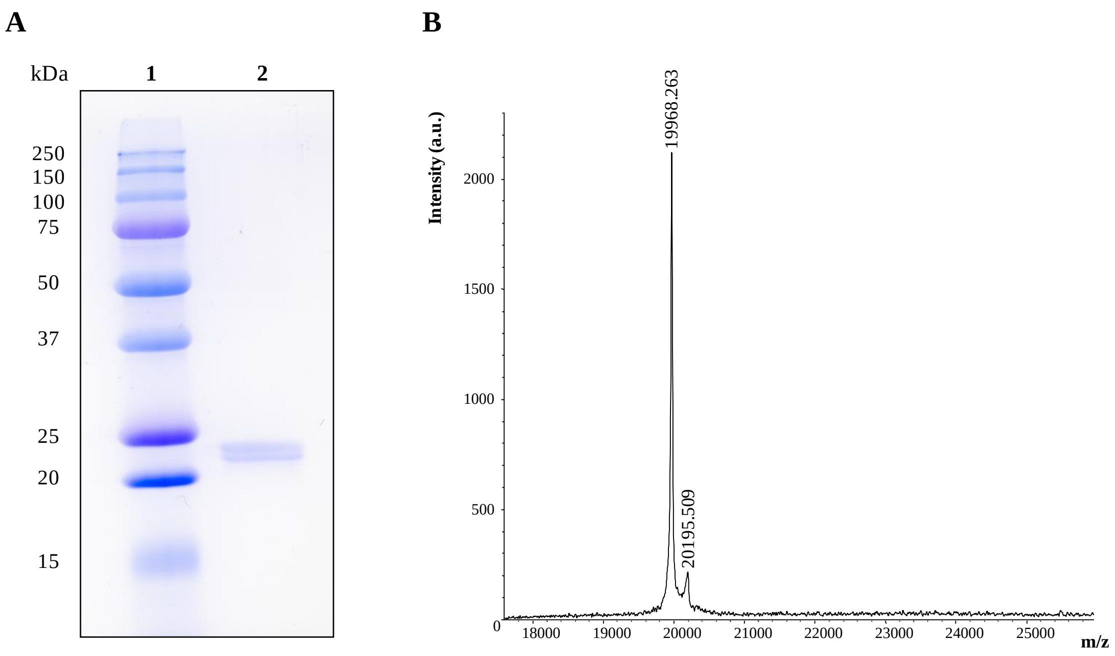

Figure S1. A) SDS-PAGE analysis of isolated rXynM. B) Accurate determination of the molecular mass of rXynM by MALDI-TOF.

Table S1. Central composite experimental design for optimizing the synthesis of 2-(6-hydroxynaphthyl)-β-D-xylopyranoside from beechwood xylan.

Time (min)	Temperature (°C)	pH	[rXynM] (μM)	[rBxTW1] (μM)	[Product] (mM)
10	30	2.2	5.00	0.10	0.02
10	30	2.2	5.00	1.00	0.01
10	30	6.0	5.00	1.00	0.12
10	30	6.0	5.00	0.10	0.10
10	30	2.2	1.00	0.10	0.01
10	30	2.2	1.00	1.00	0.33
10	30	6.0	1.00	1.00	0.32
10	30	6.0	1.00	0.10	0.01
10	40	4.1	12.50	0.55	1.18
10	50	2.2	5.00	0.10	0.21
10	50	6.0	5.00	0.10	0.30
10	50	6.0	5.00	1.00	1.33
10	50	2.2	5.00	1.00	1.51
10	50	2.2	1.00	0.10	0.06
10	50	6.0	1.00	0.10	0.01
10	50	6.0	1.00	1.00	0.29
10	50	2.2	1.00	1.00	0.81
35	30	4.1	12.50	0.55	1.02

35	30	4.1	0.50	0.55	0.55
35	40	4.1	12.50	0.55	1.57
35	40	4.1	12.50	0.55	2.08
35	40	4.1	12.50	0.55	1.90
35	40	4.1	12.50	0.10	0.61
35	40	4.1	12.50	0.55	1.39
35	40	6.0	12.50	0.55	1.80
35	40	4.1	12.50	0.55	1.64
35	40	4.1	12.50	0.55	1.52
35	40	4.1	5.00	0.55	0.85
35	40	4.1	12.50	0.55	1.74
35	40	2.2	12.50	0.55	1.73
35	40	4.1	12.50	1.00	1.63
35	40	4.1	12.50	0.55	1.38
35	40	4.1	1.00	0.55	1.41
35	40	4.1	0.50	0.55	0.91
35	40	4.1	0.50	0.55	0.95
35	40	4.1	0.00	0.55	0.03
35	40	4.1	0.00	0.00	0.00
35	40	4.1	0.00	0.00	0.00
35	40	4.1	0.00	0.55	0.08
35	40	4.1	0.00	0.55	0.04
35	50	4.1	12.50	0.55	1.61
60	30	6.0	5.00	1.00	0.85
60	30	6.0	5.00	0.10	0.35
60	30	2.2	5.00	0.10	0.41
60	30	2.2	5.00	1.00	1.22
60	30	6.0	1.00	1.00	1.46
60	30	6.0	1.00	0.10	0.30
60	30	2.2	1.00	0.10	0.31
60	30	2.2	1.00	1.00	0.53
60	40	4.1	12.50	0.55	1.55
60	50	4.1	12.50	1.00	0.87
60	50	4.1	12.50	1.00	1.19
60	50	4.1	12.50	1.00	0.87
60	50	2.4	12.50	1.00	1.35
60	50	2.4	12.50	1.00	1.32
60	50	2.4	12.50	1.00	0.99
60	50	6.0	5.00	0.10	0.89
60	50	2.2	5.00	1.00	1.20
60	50	2.2	5.00	0.10	1.04
60	50	6.0	5.00	1.00	1.07
60	50	6.0	1.00	0.10	0.14
60	50	2.2	1.00	1.00	0.67
60	50	2.2	1.00	0.10	0.45
60	50	6.0	1.00	1.00	0.24

Table S2. ANOVA results for the multiparametric model using beechwood xylan.

Source	Sum of squares	Df	Mean square	F value	p-value prob > F*
**Model**	18.74	20	0.9368	7.86	<0.0001
**Residual**	5.12	43	0.1192		
**Lack of fit**	4.56	28	0.1628	4.32	0.0022
**Pure error**	0.5652	15	0.0377		
**Cor total**	23.86	63			

*Values of prob > F less than 0.0500 indicate model terms are significant

[Product] = − 4.04105 + 0.058640A + 0.216216B – 0.769990C + 8.150×10-3D + 2.43176E – 5.10×10-4AB + 2.15×10-4AC −5.95×10-4AD – 3.890×10-3AE – 3.325×10-3BC + 2.332×10-3BD – 4.88×10-4BE + 7.014×10-3CD + 5.468×10-3CE + 2.116×10-3DE – 4.09×10-4A2 – 2.277×10-3B2 + 0.104697C2 – 3.265×10-3D2 – 0.829486E2

A: time (min); B: temperature (°C); C: pH; D: [rXynM] (µM); E: [rBxTW1] (µM).

Equation S1. Quadratic model equation for the production of DHNX from beechwood xylan.

Table S3. Central composite experimental design for optimizing the synthesis of 2-(6-hydroxynaphthyl)-β-D-xylopyranoside from birchwood xylan.

Time (min)	Temperature (°C)	pH	[rXynM] (µM)	[Xylan] (g/L)	[rBxTW1] (µM)	[Product] (g/L)
5	40	4.0	31.20	40	0.45	0.89
30	30	5.0	12.40	60	0.20	0.82
30	30	3.0	50.00	60	0.70	0.60
30	50	3.0	12.40	60	0.20	1.45
30	50	3.0	50.00	20	0.20	0.42
30	30	3.0	12.40	20	0.20	0.59
30	50	5.0	12.40	20	0.20	0.65
30	50	3.0	50.00	60	0.70	0.80
30	50	5.0	12.40	60	0.70	0.81
30	30	5.0	12.40	20	0.70	0.64
30	50	3.0	12.40	20	0.20	0.53
30	50	3.0	50.00	20	0.70	0.25

30	30	5.0	12.40	20	0.20	0.74
30	50	5.0	12.40	60	0.20	1.00
30	50	5.0	50.00	60	0.20	1.41
30	30	3.0	50.00	60	0.20	0.84
30	30	5.0	12.40	60	0.70	0.88
30	30	5.0	50.00	60	0.20	0.65
30	30	5.0	50.00	20	0.20	0.51
30	30	3.0	12.40	60	0.20	0.48
30	30	5.0	50.00	60	0.70	0.68
30	50	3.0	12.40	20	0.70	0.38
30	50	5.0	50.00	60	0.70	0.93
30	30	3.0	50.00	20	0.70	0.11
30	50	3.0	50.00	60	0.20	1.40
30	30	5.0	50.00	20	0.70	0.32
30	30	3.0	12.40	60	0.70	0.52
30	50	5.0	50.00	20	0.20	1.34
30	50	3.0	12.40	60	0.70	0.66
30	30	3.0	50.00	20	0.20	0.75
30	50	5.0	50.00	20	0.70	0.17
30	30	3.0	12.40	20	0.70	0.03
30	50	5.0	12.40	20	0.70	0.13
60	50	3.0	50.00	60	0.20	1.27
60	50	3.0	50.00	60	0.10	1.20
60	50	3.0	50.00	60	0.05	0.89
60	50	3.0	50.00	60	0.025	0.56
75	40	4.0	31.20	40	0.80	0.26
75	40	4.0	31.20	40	0.45	0.26
75	40	4.0	31.20	9	0.45	0.00
75	40	4.0	31.20	40	0.45	0.50
75	40	4.0	31.20	40	0.45	0.41
75	40	4.0	1.80	40	0.45	0.57
75	40	4.0	31.20	40	0.45	0.43
75	40	4.0	31.20	40	0.45	0.41
75	40	4.0	31.20	40	0.45	0.53
75	40	4.0	31.20	40	0.45	0.47
75	40	4.0	31.20	40	0.45	0.40
75	40	4.0	31.20	40	0.45	0.51
75	40	2.2	31.20	40	0.45	0.25
75	40	5.5	31.20	40	0.45	0.50
75	40	4.0	31.20	70	0.45	1.02
75	24	4.0	31.20	40	0.45	0.72
75	40	4.0	31.20	40	0.45	0.44
75	40	4.0	60.00	40	0.45	0.23
75	55	4.0	31.20	40	0.45	0.90
120	30	5.0	12.40	60	0.20	1.11
120	50	5.0	12.40	20	0.70	0.06
120	30	5.0	50.00	60	0.70	0.74
120	30	3.0	50.00	60	0.70	0.24

120	50	5.0	50.00	60	0.70	0.79
120	50	5.0	50.00	60	0.20	1.74
120	50	3.0	12.40	60	0.20	1.13
120	30	5.0	50.00	60	0.20	1.11
120	30	3.0	12.40	60	0.70	0.18
120	30	5.0	12.40	20	0.70	0.09
120	30	3.0	50.00	20	0.20	0.23
120	30	5.0	12.40	20	0.20	0.33
120	30	3.0	12.40	20	0.70	0.00
120	50	5.0	12.40	60	0.20	1.22
120	50	3.0	50.00	20	0.70	0.03
120	50	3.0	12.40	60	0.70	1.22
120	50	5.0	50.00	20	0.70	0.23
120	30	5.0	50.00	20	0.20	0.53
120	50	5.0	12.40	20	0.20	0.64
120	50	3.0	12.40	20	0.70	0.00
120	30	3.0	12.40	20	0.20	0.12
120	30	3.0	50.00	60	0.20	0.67
120	30	3.0	50.00	20	0.70	0.00
120	50	5.0	50.00	20	0.20	0.45
120	50	3.0	50.00	60	0.70	0.95
120	50	3.0	50.00	20	0.20	0.63
120	30	3.0	12.40	60	0.20	0.61
120	30	5.0	50.00	20	0.70	0.07
120	50	5.0	12.40	60	0.70	0.74
120	30	5.0	12.40	60	0.70	0.65
120	50	3.0	50.00	60	0.20	0.89
145	40	4.0	31.20	40	0.45	0.12

Table S4. ANOVA results for the multiparametric model using birchwood xylan.

Source	Sum of squares	Df	Mean square	F value	p-value prob > F*
**Model**	10.70	27	0.3964	9.24	<0.0001
**Residual**	2.57	60	0.0429		
**Lack of fit**	2.52	51	0.0495	8.68	0.0008
**Pure error**	0.0513	9	0.0057		
**Cor total**	13.28	87			

*Values of prob > F less than 0.0500 indicate model terms are significant

$$[\text{Product}] = + 2.62625 – 0.012671A – 0.131701B + 0.237935C – 4.530\times10\text{-}3D + 2.20900E – 0.022099F + 4.6\times10\text{-}5AB + 4.49\times10\text{-}4AC + 7.21157\times10\text{-}8AD – 4.52\times10\text{-}4AE + 6.8\times10\text{-}5AF – 3.809\times10\text{-}3BC + 7.8\times10\text{-}5BD − 0.013789BE + 3.81\times10\text{-}4BF + 6.82\times10\text{-}4CD − 0.107926CE + 3.68\times10\text{-}4CF − 4.412\times10\text{-}3DE − 1.10132\times10\text{-}6DF + 5.507\times10\text{-}3EF + 3.3\times10\text{-}5A2 + 1.790\times10\text{-}3B2 – 2.960\times10\text{-}3C2 + 2.3\times10\text{-}5D2 – 2.14132E2 + 1.35\times10\text{-}4F2$$

A: time (min); B: temperature (°C); C: pH; D: [rXynM] (μM); E: [rBxTW1] (μM); F: [xylan] (g/L).

Equation S2. Quadratic model equation for the production of DHNX from birchwood xylan.

NMR

Table S5. Chemical shift data from 2-(6-hydroxynaphthyl) β-D-xylopyranoside.

	**DHNX**	
	**^{1}H (ppm)**	**^{13}C (ppm)**
**1**	5.09	101.15
**2**	3.51	73.64
**3**	3.51	75.58
**4**	3.65	69.29
**5 (axial)**	3.44	65.04
**5 (equatorial)**	3.97	
**Naphthalene ring**	7.11	118.96
	7.20	109.14
	7.22	118.86
	7.40	111.62
	7.69	128.07
	7.73	129.23

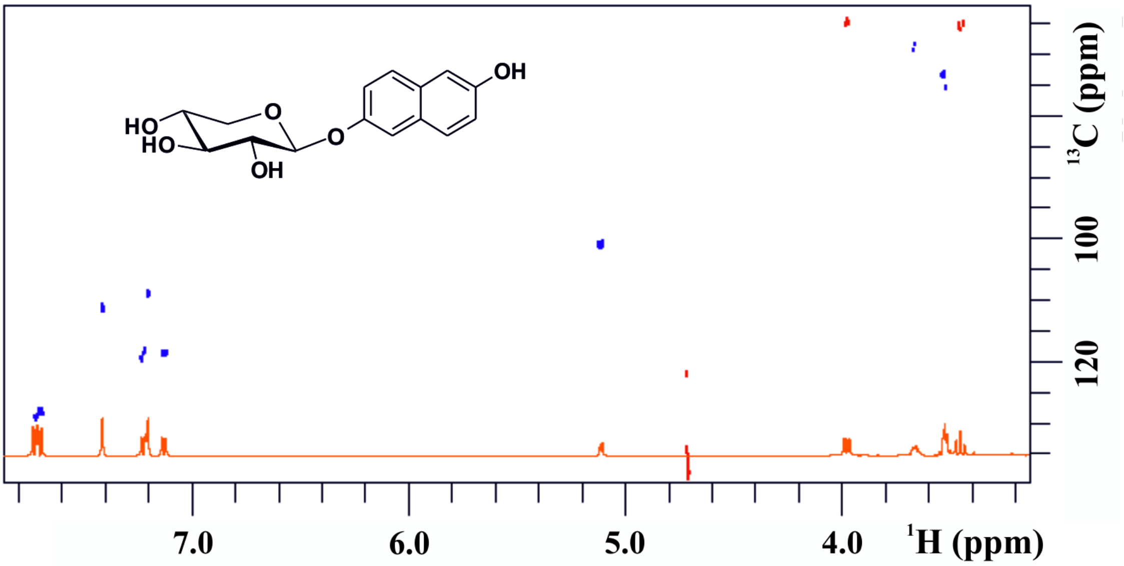

Figure S2. ^{1}H-^{13}C HSQC edited spectra (blue, CH/CH_3; red, CH_2) and ^{1}H 1D spectra (orange) of DHNX obtained from beechwood xylan.

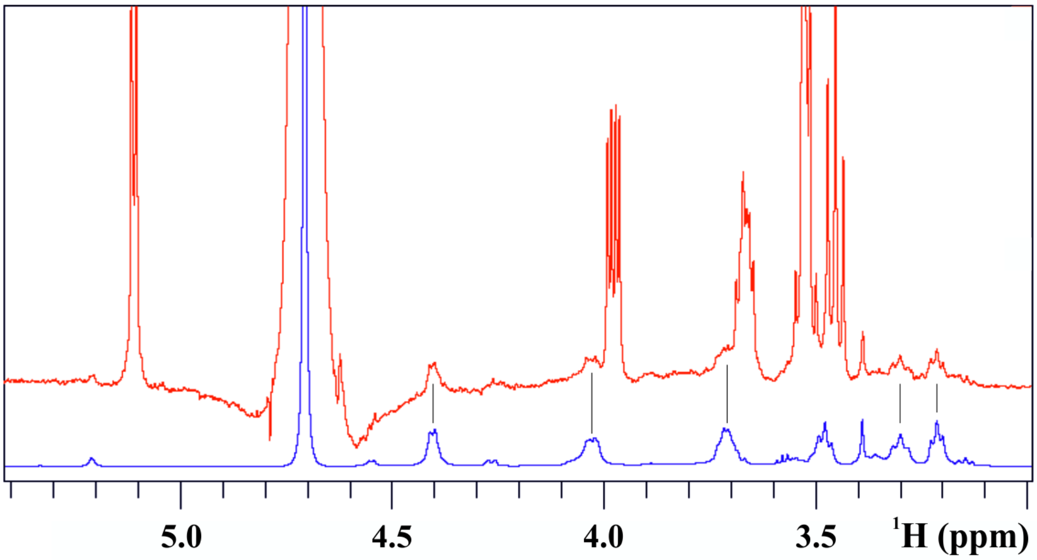

Figure S3. Superimposition of the ^{1}H 1D spectra of the beechwood xylan substrate (blue) and the DHNX obtained (red). The raw beechwood background is observed in the DHNX sample. The ratio of integrals of highlighted non-overlapping peaks was employed to estimate the purity.

Mass spectrometry

Mass spectra (negative mode) of the isolated DHNX from beechwood and birchwood are displayed below. The identified product adducts are appropriately labeled.
