# Supplementary material for: Exploiting xylan as sugar donor for the synthesis of an antiproliferative xyloside using an enzyme cascade
Source: Microb Cell Fact. 2019 Oct 10;18:174. doi: 10.1186/s12934-019-1223-9 (PMC6788083; doi:10.1186/s12934-019-1223-9)
Supplement: Supplementary file 2 — Additional file 2. Mass spectrometry. Mass spectra (negative mode) of the isolated DHNX from beechwood and birchwood are displayed below. The product adducts identified are appropriately labeled. [file 12934_2019_1223_MOESM2_ESM.pdf]

ADDITIONAL FILE 2

MASS SPECTROMETRY

Mass spectra (negative mode) of the isolated DHNX from beechwood and birchwood are displayed below. The product adducts identified are appropriately labeled.

Mass Spectrum List Report

Acquisition Date	9/4/2017 9:13:17 AM
Operator	Dr Kayali
Instrument	HCTultra PTM Discovery System

Acquisition Parameter

Ion Source Type	ESI	Ion Polarity	Negative	Alternating Ion Polarity	off
Mass Range Mode	Ultra Scan	Scan Begin	100 m/z	Scan End	1200 m/z
Capillary Exit	-9.0 Volt	Skimmer	-40.0 Volt	Trap Drive	35.0
Accumulation Time	51778 µs	Averages	8 Spectra	Auto MS/MS	off

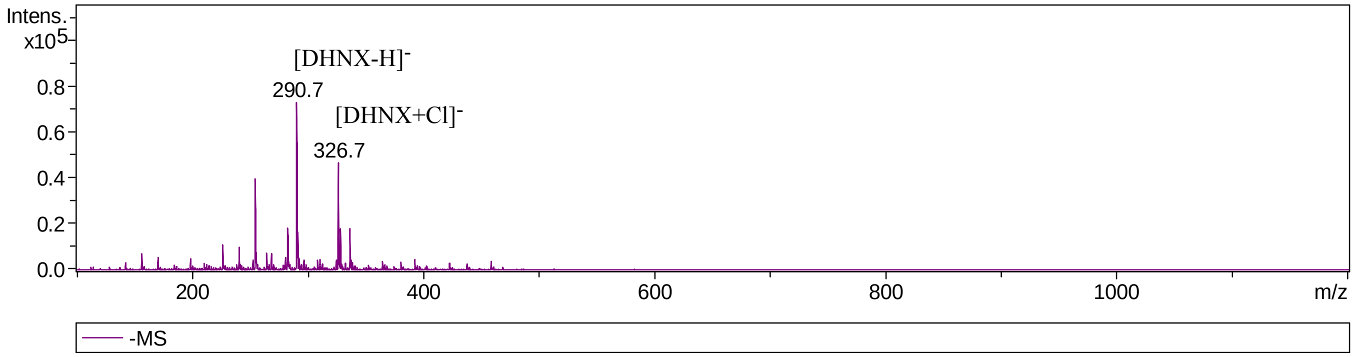

#	m/z	I
1	142.8	3190
2	156.8	7070
3	170.8	5544
4	184.8	1936
5	198.8	4840
6	210.7	2946
7	212.8	2308
8	226.8	11025
9	238.7	2223
10	240.8	9786
11	241.8	2235
12	252.8	4208
13	254.9	39763
14	255.8	7518
15	256.8	2433
16	264.8	7474
17	266.8	2320
18	268.8	7178
19	270.8	2274
20	278.8	2013
21	280.9	5417
22	282.9	18324
23	283.9	3581
24	284.8	2402
25	290.7	72753
26	291.6	16655
27	292.7	5134
28	294.7	2411
29	296.8	4338
30	298.8	2397
31	308.8	4252
32	310.8	4669
33	312.8	2715
34	324.8	4322
35	326.7	46804
36	327.6	9375

Mass Spectrum List Report

#	m/z	I
37	328.7	17861
38	329.6	2716
39	332.7	2817
40	336.7	18089
41	337.7	4325
42	338.8	3186
43	352.8	2107
44	364.8	3800
45	366.9	2275
46	380.7	3333
47	392.9	4591
48	422.7	2956
49	437.7	2586
50	458.7	3634

Mass Spectrum List Report

Analysis Info

Analysis Name	D:\Data\201811\DHNX0000.d	Acquisition Date	11/15/2018 12:59:11 PM
Method	alberto01.m	Operator	Dr Kayali
Sample Name	DHNX	Instrument	HCTultra PTM Discovery System
Comment	directo en LIQUIDO+ MeOH		

Acquisition Parameter

Ion Source Type	ESI	Ion Polarity	Negative	Alternating Ion Polarity	off
Mass Range Mode	Std/Enhanced	Scan Begin	50 m/z	Scan End	1000 m/z
Capillary Exit	-93.2 Volt	Skimmer	-40.0 Volt	Trap Drive	43.2
Accumulation Time	1744 µs	Averages	8 Spectra	Auto MS/MS	off

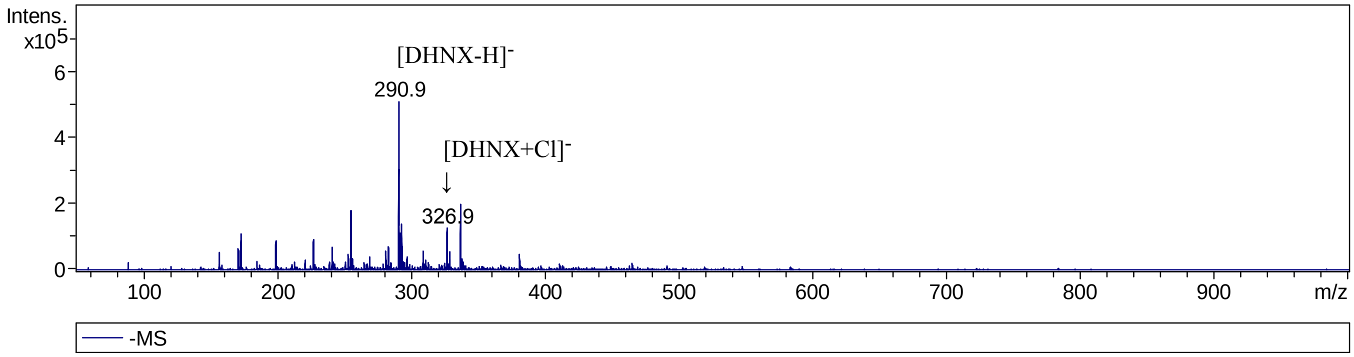

#	m/z	I
1	88.9	22665
2	120.9	11201
3	142.9	8371
4	156.9	53898
5	158.9	14375
6	171.0	65025
7	171.9	7717
8	173.0	109439
9	176.9	8350
10	185.0	26271
11	186.9	13489
12	199.0	87345
13	200.0	10035
14	211.0	16997
15	213.0	23170
16	214.0	7768
17	215.0	7724
18	220.9	30024
19	225.0	12554
20	227.1	92269
21	228.0	15583
22	228.9	8715
23	235.0	10614
24	239.0	23882
25	241.0	68727
26	242.0	24815
27	243.0	18162
28	250.9	24558
29	253.1	47065
30	254.1	8983
31	255.1	178963
32	256.0	34069
33	257.0	12242
34	265.0	22131
35	267.0	18500
36	269.1	39413

#	m/z	I
37	270.0	9083
38	272.9	8964
39	275.0	7655
40	279.0	18956
41	281.1	57456
42	282.0	13671
43	283.1	69609
44	284.0	10164
45	285.0	22060
46	288.9	8453
47	290.9	509197
48	291.9	110376
49	293.0	137984
50	294.0	26819
51	295.0	22286
52	297.0	39378
53	298.0	8068
54	298.9	16007
55	300.9	11930
56	302.9	8968
57	304.9	7948
58	306.9	10858
59	309.0	57012
60	310.0	17623
61	311.0	30151
62	312.0	8220
63	313.0	21460
64	315.0	10689
65	321.0	15461
66	323.0	15019
67	325.0	20614
68	325.9	8266
69	326.9	127458
70	327.9	18957
71	328.9	55631
72	329.9	8341
73	336.9	197658
74	337.9	34152
75	338.9	24390
76	340.0	11394
77	340.9	12891
78	350.9	9901
79	353.0	10866
80	360.9	8237
81	367.1	14055
82	369.0	11002
83	373.0	7880
84	380.9	48038
85	381.9	12696
86	395.1	8957
87	397.0	12504
88	403.0	9251
89	410.9	17451
90	412.9	12341
91	413.9	8561
92	425.1	7775
93	445.8	8279
94	449.0	10004
95	463.0	12386
96	465.0	19343
97	469.0	7731
98	490.9	12733
99	547.2	9581
100	583.0	7689
